# A Cocktail of Three Virulent Phages Controls Multidrug-Resistant *Salmonella* Enteritidis Infection in Poultry

**DOI:** 10.3389/fmicb.2022.940525

**Published:** 2022-07-06

**Authors:** Yue Li, Peilin Lv, Deshi Shi, Hongze Zhao, Xu Yuan, Xiue Jin, Xiliang Wang

**Affiliations:** ^1^Key Laboratory of Agricultural Microbiology, College of Veterinary Medicine, Huazhong Agricultural University, Wuhan, China; ^2^College of Veterinary Medicine, Huazhong Agricultural University, Wuhan, China; ^3^Hubei Provincial Institute of Veterinary Drug Control, Wuhan, China

**Keywords:** *Salmonella*, virulent phage, multi-drug resistance, genomic, phage cocktail therapy, poultry

## Abstract

*Salmonella enterica* is not only the most common pathogen of poultry and poultry-derived products but is also a significant foodborne pathogen. In recent years, many *S. enterica* isolates have exhibited multi-drug resistance, which places huge pressure on global economy and health. Since phages are an attractive alternative to biocontrol pathogens, we isolated a total of 15 *Salmonella* phages from sewage effluent, sediment, and chicken manure. The GRNsp1, GRNsp3, GRNsp6, GRNsp21, GRNsp27, GRNsp30, GRNsp50, and GRNsp51 phages exhibited a wide host range against *S. enterica* serovars Enteritidis and Typhimurium *in vitro*. In particular, GRNsp51 exerted highly efficient lytic effects against a large proportion of *S.* Enteritidis and *S.* Typhimurium strains isolated from different regions of China. Meanwhile, GRNsp8 expanded the host range of GRNsp6 and GRNsp51. Based on their host ranges and lytic capacities, GRNsp6, GRNssp8, and GRNsp51 were selected for further investigation. Morphology, one-step growth curves, and stability assays revealed that GRNsp6, GRNsp8, and GRNsp51 all belong to the *Caudovirales* order and display relatively short latency periods with broad pH and thermal stability. Genomic analysis indicated that the genomes of these three phages contained no genes related to virulence, antibiotic resistance, or lysogeny. In addition, we tested the effectiveness of a cocktail composed of these three phages against *S.* Enteritidis in a chicken model. Treatment with the oral phage cocktail 24 h before or alongside *Salmonella* challenge significantly reduced colonization of the intestinal tract and decreased the mRNA expression of IL-6, IFN-γ, and IL-1β in the duodenum. Together, these findings indicate that a cocktail of the GRNsp6, GRNsp8, and GRNsp51 phages could serve as an effective antimicrobial therapeutic agent against multidrug-resistant *Salmonella* in animal production to mitigate infections by multiple zoonotic *Salmonella* species.

## Introduction

*Salmonella enterica* is one of the most important food-borne pathogens as it not only infects chickens, pigs, and other animals, but also poses a serious threat to human health. Salmonellosis is common worldwide, with around 52702 confirmed cases reported in 2020 in the EU, accounting for 22.5% of all foodborne outbreaks in humans (European Food Safety Authority and European Centre for Disease Prevention and Control, 2021). The severity of disease caused by *Salmonella* infection varies and mainly depends on host factors and the *Salmonella* serovar (Threlfall, 2002). Around 2000 distinct *Salmonella enterica* serovars have been reported, among which *S.* Typhi and *S.* Paratyphi are strict human pathogens, whereas other serovars are specifically related to animals (LeLièvre et al., 2019). Human infections are usually associated with the consumption of contaminated foods and water, direct contact with infected animals, or the consumption of food made from infected animals (Hoffmann and Scallan, 2017). In particular, poultry, pigs, and cattle are considered to be animal reservoirs of *Salmonella* infection in humans (Threlfall, 2002; European Food Safety Authority and European Centre for Disease Prevention and Control, 2021).

The extensive use and abuse of antimicrobial agents in animal production in recent decades has led to the development of widespread antimicrobial resistance (AMR). This has reduced the efficacy of common antimicrobials and increased the severity, morbidity, and mortality of salmonellosis in humans and animals. Due to the lack of new candidate antibiotic compounds in the development pipeline, AMR has made the prevention and control of *Salmonella* infection increasingly challenging. In addition, the successive prohibition of many antibiotics and growth promoters for broiler chicken husbandry in the EU and US has made it very difficult and expensive to maintain biosecurity in poultry farms (Dibner and Richards, 2005; Seal et al., 2013). Safe, effective, and cost-effective antibiotic substitutes are therefore urgently required to control *Salmonella* infections in animals and foodstuffs.

Bacteriophages are promising biocontrol agents with numerous advantages, including self-replication abilities, biosphere richness, specificity and sensitivity against selected bacteria without disturbing natural microbiota, and the ability to potentially kill multidrug-resistant (MDR) cells (Thurber, 2009). In addition, bacteriophages are relatively safe as they exist as commensal organisms in humans (Melo and Oliveira, 2020; Petrovic Fabijan et al., 2020). Numerous studies have reported that phages can reduce the number of target bacteria and successfully control bacteria in animals with natural or simulated infection (Bardina et al., 2012; Huang et al., 2018; Bao et al., 2020; Li et al., 2020). Thus, bacteriophages could be a reasonable alternative to antibiotics for sustainable animal production.

Despite their immense potential, phages must be investigated and characterized in detail before safe and effective phage therapies can be developed. Firstly, a large repository of phages that infect various bacterial species and strains should be established to identify phages with high lytic capacity capable of treating specific diseases caused by different bacteria in humans and animals. Secondly, the therapeutic phages should be virulent and free of genes related to pathogenicity, lysogeny, and toxins. Finally, the selected phage must be stable and easy to prepare in order to limit industrial production costs and its therapeutic efficacy and safety must be fully evaluated through numerous clinical trials (Sulakvelidze et al., 2001; Grant et al., 2016; Górski et al., 2019).

In this study, we isolated a variety of lytic phages and screened the ability of those with predicted broad-spectrum activity to control various drug-resistant *Salmonella* strains isolated from China. A cocktail of the selected phages was developed and its therapeutic potential against *S. enterica* infection was evaluated in chickens. Thus, our study introduces new candidate phages and provides guidance for the development of safe and effective anti-*Salmonella* therapies.

## Materials and Methods

### Bacterial Strains and Growth Conditions

The details of all *Salmonella* strains used in this study are listed in Table 1. Standard strains were purchased from the American Type Culture Collection (ATCC, Gaithersburg, MD, United States) and the National Centre for Medical Culture Collections (CMCC, Beijing, China). Other strains were isolated from poultry, swine, and environmental sewage, and were preserved in 25% (v/v) glycerol at –80°C and cultured in tryptone soya broth (TSB; Becton Dickinson, Sparks, MD, United States) or tryptone soya agar (TSA; Becton Dickinson, Sparks, MD, United States) at 37°C. A total of 88 *Salmonella* strains were used to determine the lytic range of phages, including the most prevalent serovars recovered from livestock and poultry clinical samples between 2015 and 2021. *S.* Enteritidis ATCC 13076, *S.* Enteritidis ATCC13311, and *S.* Typhimurium CMCC50115 were selected for phage isolation, propagation, and purification. The serovars of all clinical isolates were characterized using whole genome sequencing (WGS) performed by ANNOROAD Gene Technology Co., Ltd (Beijing, China). AMR identification was performed using the disk diffusion method (Bauer et al., 1966).

**TABLE 1:** Bacterial strains used in this study.

Bacterial strain	Year	Serovar	ST	Serogroup	Isolation source	Regions^b^	Antibiotic resistance^a^	
							Resistance	Intermediate
ATCC13076		Enteritidis						
GDC200607T	2020	Enteritidis	11	D1	Chicken fecal	GD	SF, S, NA	CRO, CIP, SF
GDC200608B	2020	Enteritidis	11	D1	Chicken fecal	GD	SF, S, K, NA	CN, CRO, CIP, SF
GDC200613	2020	Enteritidis	11	D1	Chicken fecal	GD	SF, S, K, CN, NA	CRO, CIP
GDC200614	2020	Enteritidis	11	D1	Chicken fecal	GD	TE, SF, S, K, CN, AMP, NA	CRO, CIP
GXC180601	2018	Enteritidis	11	D1	Chicken fecal	GX	SF, S, K, CN, AMP, NA	CRO, CIP
GXC190301	2019	Enteritidis	11	D1	Chicken fecal	GX	TE, SF, S, K, AMP, NA	CN, CRO, CIP
GXC200501	2020	Enteritidis	11	D1	Chicken fecal	GX	TE, SF, S, K, CN, AMP, NA, CIP	FOX, CRO
GXC200714	2020	Enteritidis	11	D1	Chicken fecal	GX	TE, SF, S, AMP, NA	K, CRO, CIP
GXC200715	2020	Enteritidis	11	D1	Chicken fecal	GX	TE, SF, S, AMP, NA	CIP
GXC200717	2020	Enteritidis	11	D1	Chicken fecal	GX	TE, SF, S, AMP, NA	CIP
HENC170401	2017	Enteritidis	11	D1	Chicken fecal	HEN	SF, S, AMP, NA, CIP	K, CN
HENC170402	2017	Enteritidis	11	D1	Chicken fecal	HEN	SF, S, AMP, NA, CIP	K, CN
HENC210401	2021	Enteritidis	11	D1	Chicken fecal	HEN	SF, S, K, CN, AMP, CRO, NA, CIP	FOX
HENC210402	2021	Enteritidis	11	D1	Chicken fecal	HEN	SF, S, K, CN, AMP, CRO, NA, CIP	FOX
HENC210403	2021	Enteritidis	11	D1	Chicken fecal	HEN	SF, S, K, CN, AMP, CRO, NA, CIP	FOX
SDC170403	2017	Enteritidis	11	D1	Chicken fecal	SD	SF, S, AMP, CRO, NA, CIP	K, CN
SDC170404	2017	Enteritidis	11	D1	Chicken fecal	SD	SF, S, AMP, CRO, NA, CIP	K, CN
SDC170405	2017	Enteritidis	11	D1	Chicken fecal	SD	SF, S, K, CN, AMP, CRO, NA, CIP	
SDC190802	2019	Enteritidis	11	D1	Chicken fecal	SD	TE, SF, S, K, CN, AMP, NA, CIP	AMC, CRO
SDC190803	2019	Enteritidis	11	D1	Chicken fecal	SD	TE, SF, S, K, CN, AMP, NA, CIP	AMC, CRO
SDC211101	2011	Enteritidis	11	D1	Chicken fecal	SD	TE, SF, S, AMP, FOX, NA	CRO, CIP
JSC160601B	2016	Enteritidis	11	D1	Chicken fecal	JS	SF, S, K, CN, AMP, CRO, NA, CIP	FOX
JSC160602	2016	Enteritidis	11	D1	Chicken fecal	JS	SF, S, CRO, NA, CIP	
JSC160603	2016	Enteritidis	11	D1	Chicken fecal	JS	TE, SF, S, K, CN	NA, CIP
JSC190602	2019	Enteritidis	11	D1	Chicken fecal	JS	SF, S, CIP	K, CN, NA
CMCC50115		Typhimurium	19	B	CMCC			
ATCC13311		Typhimurium	2066	B	ATCC			
GDC190801	2019	Typhimurium	19	B	Chicken fecal	GD	SF, S, K, CN, NA, CIP	FOX, CRO
HENC191101	2019	Typhimurium	19	B	Chicken fecal	HEN	SF, S, K, CN, NA, CIP	
SDC200701	2020	Typhimurium	19	B	Chicken fecal	SD	SF, S, K, CN, CRO, NA, CIP	FOX
SDC200801	2020	Typhimurium	19	B	Chicken fecal	SD	SF, S, NA	CIP
HBC200706	2020	Typhimurium	19	B	Chicken fecal	HB	SF, S, K, CN, CIP	CRO, NA
HBC150901	2015	Typhimurium	19	B	Chicken fecal	HB	SF, S	K, CIP
HBC150902	2015	Typhimurium	19	B	Chicken fecal	HB	SF, S	K, CIP
HBC190901	2019	Typhimurium	19	B	Chicken fecal	HB	TE, SF, S, K, CN, AMP, NA, CIP	
HBP191102	2019	Typhimurium	19	B	Swine fecal	HB	TE, C, FON, SF, S, AMP	K, CN, CIP
HBP191103	2019	Typhimurium	19	B	Swine fecal	HB	TE, C, FON, SF, S, K, AMP, CIP	CN, NA
HBP210708	2021	Typhimurium	34	B	Swine fecal	HB	TE, C, FON, SF, S, K, CN, AMP, NA, CIP	CRO
GXC180602	2018	I 1,4,[5],12:i:-	34	B	Chicken fecal	GX	TE, C, FON, SF, S, K, CN, AMP, CRO, NA, CIP	AMC
GXC180603	2018	I 1,4,[5],12:i:-	34	B	Chicken fecal	GX	TE, C, FON, SF, S, K, CN, AMP, CRO, NA, CIP	AMC
HBP190303	2019	I 1,4,[5],12:i:-	34	B	Swine fecal	HB	TE, C, FON, SF, S, K, CN, AMC, AMP, CRO, NA, CIP	FOX
HBP190801	2019	I 1,4,[5],12:i:-	34	B	Swine fecal	HB	SF, S, AMP, CRO	K, AMC, CIP
HBP190802	2019	I 1,4,[5],12:i:-	34	B	Swine fecal	HB	TE, C, FON, SF, S, K, AMP	CRO, NA, CIP
HBP191001	2019	I 1,4,[5],12:i:-	34	B	Swine fecal	HB	TE, C, FON, SF, S, K, CN, AMP, CRO	CIP
HBP191002	2019	I 1,4,[5],12:i:-	34	B	Swine fecal	HB	TE, C, FON, SF, S, K, CN, AMP, CRO	NA, CIP
HBP191101	2019	I 1,4,[5],12:i:-	34	B	Swine fecal	HB	TE, C, FON, SF, S, K, CN, AMC, AMP, CRO, NA, CIP	FOX
CVCC1791		Gallinarum	92	D1	CVCC			
HENC161001	2016	Gallinarum	92	D1	Chicken fecal	HEN	TE, SF, S, NA	CIP
HENC161002	2016	Gallinarum	92	D1	Chicken fecal	HEN	TE, SF, S, NA, CIP	CRO
SDC170410	2017	Gallinarum	92	D1	Chicken fecal	SD	SF, S, AMP, CRO, NA, CIP	AMC
GXC200706	2020	Gallinarum	92	D1	Early-dead embryos of chickens	GX	SF, S, AMP, NA, CIP	
GDC200604	2020	Gallinarum	2151	D1	Chicken fecal	GD	TE, C, FON, SF, S, NA, CIP	
GDC200607	2020	Gallinarum	2151	D1	Chicken fecal	GD	SF, AMP, NA, CIP	S
GDC200609	2020	Gallinarum	2151	D1	Chicken fecal	GD	TE, C, FON, SF, S, AMP, NA, CIP	
GDC200615	2020	Gallinarum	2151	D1	Chicken fecal	GD	TE, C, FON, SF, S, AMC, AMP, FOX, CRO, NA, CIP	
HNC200501	2020	Gallinarum	78	D1	Chicken fecal	HN	SF, S	NA, CIP
HNC200503	2020	Gallinarum	78	D1	Chicken fecal	HN	SF, S	NA, CIP
HBP190301	2019	Derby	40	B	Swine fecal	HB	TE, C, FON, SF, S, K, CN, AMP, CRO, NA, CIP	AMC
HBP190302	2019	Derby	40	B	Swine fecal	HB	TE, C, FON, SF, S, K, CN, AMP, NA, CIP	AMC, CRO
HBP190401	2019	Derby	40	B	Swine fecal	HB	TE, C, FON, SF, S, K, CN, AMP, NA, CIP	AMC, CRO
HBP190402	2019	Derby	40	B	Swine fecal	HB	TE, C, FON, SF, S, K, CN, AMP, CIP	AMC, CRO, NA
HBP190403	2019	Derby	40	B	Swine fecal	HB	C, FON, SF, K, CN, AMP, NA, CIP	TE, S, AMC, CRO
HBP190404	2019	Derby	40	B	Swine fecal	HB	C, FON, SF, S, K, CN, AMP, NA, CIP	TE, AMC, CRO
HBP190701	2019	Derby	40	B	Swine fecal	HB	TE, C, FON, SF, S, K, CN, AMP, NA, CIP	AMC, CRO
GXC200710	2020	Weltevreden	365	E1	Chicken fecal	GX	SF	S
GXC200711	2020	Weltevreden	365	E1	Chicken fecal	GX	SF	S
GXC200903	2020	Senftenberg	14	E4	Chicken fecal	GX	TE, C, FON, SF, S, K, CN, AMP, CRO, CIP	NA
GXC200905	2020	Senftenberg	14	E4	Chicken fecal	GX	TE, C, FON, SF, S, K, CN, AMP, CRO, CIP	NA
GXC200901	2020	Thompson	26	C1	Chicken fecal	GX	TE, C, FON, SF, S, K, CN, AMP, CRO, CIP	NA
GXC200902	2020	Thompson	26	C1	Chicken fecal	GX	TE, C, FON, SF, S, K, CN, AMP, CRO, CIP	NA
GXC200904	2020	Thompson	26	C1	Chicken fecal	GX	TE, C, FON, SF, S, K, CN, AMP, CRO, CIP	NA
GXC190801	2019	Kentucky	198	C2-C3	Chicken fecal	GX	TE, C, FON, SF, S, K, CN, AMP, CRO, NA, CIP	AMC
HBP210705	2021	Kentucky	198	C2-C3	Swine fecal	HB	TE, C, FON, SF, S, K, CN, AMP, CRO, NA, CIP	
HBP210706	2021	Kentucky	198	C2-C3	Swine fecal	HB	TE, C, FON, SF, S, K, CN, AMP, CRO, NA, CIP	
HBP210707	2021	Kentucky	198	C2-C3	Swine fecal	HB	TE, C, FON, SF, S, K, CN, AMP, CRO, NA, CIP	
SDC211102	2011	Kentucky	198	C2-C3	Chicken fecal	SD	TE, SF, S, K, CN, AMP, CRO, NA, CIP	
HBP210709	2021	Indiana	17	B	Swine fecal	HB	TE, C, FON, SF, S, K, CN, AMP, CRO, NA, CIP	K, CN
CMCC47001		Arizonae	106	–	CMCC			
GXC202106	2021	Javiana	24	D1	Chicken fecal	GX	SF, S, K, CN, CRO	AMP, FOX, NA, CIP
GXC200709	2020	Braenderup	22	C1	Chicken fecal	GX	SF, S, NA	
HBW210702	2021	IV O:57:z4, z32:-	433	O:57	Ambient sewage	HB	SF, S, AMC, AMP, CRO	K, CIP
CMCC50071		Typhi	1	D1	CMCC			
CMCC50093		Paratyphi A	85	A	CMCC			
CMCC50094		Paratyphi B	86	B	CMCC			
HBW210705	2021	Paratyphi B	86	B	Ambient sewage	HB	SF, S, K, CIP	CN
HBW210703	2021	Paratyphi B	86	B	Ambient sewage	HB	SF, S, K, CIP	CN
HBW210704	2021	Paratyphi B	86	B	Ambient sewage	HB	SF, S, CIP	K, CN

*^a^Disk diffusion test was used to test resistance to the following antibiotics: tetracycline (TE), florfenicol (FON), chloramphenicol (C), sulfisoxazole (SF), streptomycin (S), kanamycin (K), gentamicin (CN), amoxicillin/clavulanic acid (AMC), ampicillin (AMP), cefoxitin (FOX), ceftriaxone (CRO), nalidixic acid (NA), and ciprofloxacin (CIP).*

*^b^GD, Guangdong; GX, Guangxi; HEN, Henan; SD, Shandong; JS, Jiangsu; HB, Hubei; HN, Hunan.*

### Bacterial Genome Library Preparation, Sequencing, and Analysis

Genomic DNA was extracted from isolates using a Bacterial DNA Kit (Omega Bio-tek, Norcross, GA, United States). Bacterial genomes were sequenced using an Illumina NovaSeq 6000 platform (Illumina, San Diego, CA, United States) with 2*150 bp paired-end reads and were assembled using SPAdes v3.14.1 (Bankevich et al., 2012). Genome annotation was performed using Prokka (v1.14.6) (Seemann, 2014), which uses Prodigal (v2.6.3) (Hyatt et al., 2010) to identify protein-coding genes. *Salmonella* serovars were predicted using the *Salmonella in silico* Typing Resource (SISTR) tool^1^ (Yoshida et al., 2016). Sequence typing (ST) were performed using SRST2 v0.2.0 (Inouye et al., 2014). The 88 *Salmonella* sequences reported in this paper have been deposited in the National Center for Biotechnology Information BioProject database (NCBI, BioProject ID: PRJNA844535).

### Antimicrobial Susceptibility Testing

The antimicrobial susceptibility (Table 1) of each *Salmonella* isolate was determined using the agar-disk diffusion susceptibility method (Bauer et al., 1966) according to criteria published by the Clinical and Laboratory Standards Institute (Weinstein et al., 2020). Thirteen antimicrobials were tested: tetracycline (TE; 30 μg), florfenicol (FON; 30 μg), chloramphenicol (C; 30 μg), sulfisoxazole (SF; 300 μg), streptomycin (S; 10 μg), kanamycin (K; 30 μg), gentamicin (CN; 10 μg), amoxicillin/clavulanic acid (AMC; 30 μg), ampicillin (AMP; 10 μg), cefoxitin (FOX; 30 μg), ceftriaxone (CRO; 30 μg), nalidixic acid (NA; 30 μg), and ciprofloxacin (CIP; 5 μg).

### Bacteriophage Isolation and Propagation

Sewage effluent, sediment, and manure samples were taken from farms in different regions. Solid particles were removed from water samples by centrifugation at 10,000 × *g* for 15 min and cellular microorganisms were excluded using a 0.22 μm (Merck Millipore, Cork, Ireland) sterile filter. Sediment and chicken manure samples were first dissolved in 10 mL TSB and then treated in the same way as the water samples. *S*. Enteritidis ATCC 13076, *S*. Enteritidis ATCC13311, and *S*. Typhimurium CMCC50115 were used to enrich the bacteriophages using a modified version of the method described by Kim et al. (2019) and Kwon et al. (2020), as the serovars represented by these strains are highly prevalent in human salmonellosis (European Food Safety Authority [EFSA], 2010; European Food Safety Authority and European Centre for Disease Prevention and Control, 2021). The filtrate (5 mL) was then mixed with 5 mL double-strength TSB pre-inoculated with 1% of overnight-cultured host strain and incubated at 37°C in a shaker at 160 rpm for 12 h. After the mixture had been centrifuged at 8000 rpm and filtered, phage presence was verified using the conventional double-layer agar method (Van Twest and Kropinski, 2009; Guo et al., 2021). Double-layered agar plates were incubated at 37°C for 18 ± 2 h for visualization and single plaques on the plates were purified five times to obtain a pure phage isolate (Duc et al., 2020; Shang et al., 2021). Phage propagation was performed as described previously (Lu et al., 2020).

### Host Range Determination

The host range of the isolated phages was determined using the spot assay (Kutter, 2009; Fong et al., 2017). Purified phages were normalized to a concentration of 10^9^ plaque forming unit per milliliter (PFU/mL) in SM buffer (Leagene, Beijing, China) and were spotted (5 μL) onto lawns of test *Salmonella*. The plates containing the test *Salmonella* lawns were a mixture of 200 μL bacterial broth and 3 mL 0.7% agar, which was overlaid on a plate a bottom layer of TSA. After the drops had been allowed to dry at room temperature, the plates were incubated at 37°C for 18 ± 2 h. Cell lysis zones were evaluated as described previously (Kutter, 2009), with 0 representing no lytic zone and +4 representing a completely clear zone. These values were converted into a heat map and visualized using the iTOL tool (Letunic and Bork, 2021).

### Transmission Electron Microscopy

Phages were concentrated and purified using 10% polyethylene glycol 8000 and dissolved in SM buffer as described previously (Barry and Thornsberry, 1980). Phage concentrate (5 μL, 10^9^–10^11^ PFU/mL) negatively stained with 2% (w/v) phosphotungstic acid was dropped on the carbon-coated copper grid, air-dried for 20 min, and then imaged using a Hitachi H-7600 transmission electron microscope (Tokyo, Japan) at an acceleration voltage of 80 kV.

### One-Step Growth Curves and Stability Assays

Phage one-step growth curves were measured as described previously (Duc et al., 2020). *S*. Enteritidis ATCC 13076, *S*. Enteritidis ATCC 13311, and *S*. Typhimurium CMCC50115 were used as the host and were cultured with the GRNsp6, GRNsp8, and GRNsp51 phages, respectively, at a multiplicity of infection (MOI) of 0.01 at 37°C for 140 min. During the incubation period, 100 μL of the mixture was collected at 10 or 20 min intervals to calculate phage titer using the double-layer agar assay. Phage survival at different temperatures and pH values was assessed as described previously (Shang et al., 2021). Briefly, phage suspensions (approximately 10^9^ PFU/mL) were incubated at 30, 40, 50, 60, 70, or 80°C for 1 h to test thermal stability and at pH levels from 2 to 12 for 12 h to test pH stability.

### Phage Sequencing and Genome Analysis

DNA was extracted from the isolated phages using a Viral DNA Kit (Omega Bio-tek, Norcross, GA, United States) according to the manufacturer’s instructions. Genomic DNA was sequenced on an Illumina NovaSeq 6000 platform by ANNOROAD (Beijing, China). Reads were trimmed using Trim Galore v0.6.4 (Krueger, 2012) and assembled using SPAdes v3.14.1 (Bankevich et al., 2012). Putative open reading frames (ORFs) were predicted using Rapid Annotation using Subsystem Technology v2.0 (RAST) and Heuristic GeneMarkS (Besemer and Borodovsky, 1999; Aziz et al., 2008). Functional annotation was performed using the Pfam (version 31) (Mistry et al., 2021), VOG (release 202, *n* = 26,224^2^), and EggNOG (v5.0) (Huerta-Cepas et al., 2019) databases. A circular genome map of the phage genomes was drawn using CGView (Grant and Stothard, 2008). Putative tRNA genes, virulence factors, and AMR genes were identified using tRNAscan-SE v1.3.1 (Lowe and Chan, 2016), virulence finder v2.0 (Johnson et al., 2008), and ResFinder v4.0 (Zankari et al., 2012), respectively. Toxins and genes associated with lysogenesis were screened and predicted using the NCBI^3^ and PHASTER^4^.

Whole genome sequence homology between phages was analyzed using BLASTN (NCBI, April 2022) to determine the most closely related phages (highest *E*-value and > 50% query coverage) to the isolated phages. Multiple protein sequence alignment of the terminase large subunit and major capsid protein was carried out using the clusalW algorithm with default parameters. The related phylogenetic tree was constructed and displayed in MEGA 7 using the maximum-likelihood method with 1000 bootstrap replicates. Linear whole genome comparisons were performed using Easyfig v2.2.5 (Sullivan et al., 2011). The newly characterized *Salmonella* phages GRNsp6, GRNsp8, and GRNsp51 were mixed in a 1:1:1 ratio and their efficacy was determined *in vivo*.

### Efficacy of Three-Phage Cocktail in Poultry

All animal experiments were performed in accordance with the guidelines of the China Animal Protection Association and the study was approved by the Institutional Animal Care and Use Committee of Huazhong Agricultural University (Wuhan, China) under the permit number HZAUCH-2022-0007. One-day-old *Salmonella*-free Roman-gray chickens were obtained from a commercial supplier (Laide Co., Ltd; Wuhan, China). The chickens were randomly assigned to cages (*n* = 9 per cage) and maintained in a controlled environment with food and water supplied *ad libitum*. Two chickens were euthanized prior to each experiment to confirm the absence of any preexisting *Salmonella* spp. or phages. To detect *Salmonella* in the cecum, fresh cecal digesta were collected from the chickens, weighed, and homogenized in phosphate-buffered saline (PBS). Serial dilutions were plated onto xylose lysine desoxycholate agar (Becton Dickinson, Sparks, MD, United States). When *Salmonella* could not be quantified in the cecum, it was detected using an enrichment procedure, as described previously (Bardina et al., 2012). To quantify phages in serum and cecal digesta, plaques were counted using the double-layer agar method (Van Twest and Kropinski, 2009; Guo et al., 2021). Briefly, blood samples were allowed to stand at 25°C for 2 h and centrifuged at 2000 × *g* for 15 min to separate the serum, which was serially diluted in PBS and plated onto a lawn of *S.* Enteritidis for plaque enumeration. Cecal samples were weighed, homogenized, and centrifuged at 8000 rpm for 5 min to collect supernatant for plaque enumeration. When quantitation was not possible, bacteriophage enrichment was performed, as described previously (Fiorentin et al., 2005).

To study the duration of phage cocktail activity in the cecum and serum, 3-day-old chickens (*n* = 48) were divided equally into two groups and then orally treated or injected with the phage cocktail (10^9^ PFU/animal). Four chickens were euthanized 12, 24, 48, 72, 120, and 168 h after phage inoculation. Blood and cecum samples were obtained for phage quantification.

To determine the biocontrol efficacy of the phage cocktail against MDR *S.* Enteritidis, 3-day-old chickens were orally infected with 0.1 mL *S.* Enteritidis GXC200717 (BioSample accession no. SAMN28824916), resuspended in PBS and then orally administered 0.1 mL of the phage cocktail (10^9^ PFU/animal). A total of 108 chicks were divided into four equal groups treated as described in Table 2. The chickens in groups M, C, and P were orally infected with 10^8^ CFU/animal of *S.* Enteritidis GXC200717. On days 4, 7, and 10, nine chickens per group were sacrificed and their cecal contents were collected for *Salmonella* and phage quantification. Small intestine samples were collected and total RNA was isolated (100 mg) using Trizol reagent (Invitrogen, Carlsbad, CA, United States) according to manufacturer’s instruction. Extracted RNA was quantified to 1 μg/μL and reverse transcription was performed with the Superscript reverse transcriptase (Takara, Otsu, Shiga, Japan). Cytokine gene expression levels were detected in the Bio-Rad CXF real-time PCR (Bio-Rad, Hercules, CA, United States) using iQ™ SYBR Green PCR Supermix (Takara, Otsu, Japan).

**TABLE 2:** Experimental design.

Group	Treatment schedule^a^
M	–
C	0, 1, 2, 3, 6, 9
P	–1, 0, 1, 2, 3, 6, 9
B	–

*^a^Each chicken orally administrated the phage cocktail at 10^9^ PFU/animal. –, no phage treatment; –1, phage cocktail administered 1 day before Salmonella infection.*

### Statistical Analysis

Statistical analyses were conducted using Prism Software (version 5.0, La Jolla, CA, United States). Two-way analysis of variance (ANOVA) was used for analysis of biological characteristics. Student’s *t*-test was used to determine differences between control and treatment groups. Results were expressed as mean values, with error bars indicating the standard deviations (SD). Statistical significance was defined at *p* < 0.05. *p < 0.05, ^**^p < 0.01, ^***^p < 0.001.

## Results

### Phage Isolation and Host Range Determination

**FIGURE 1:**
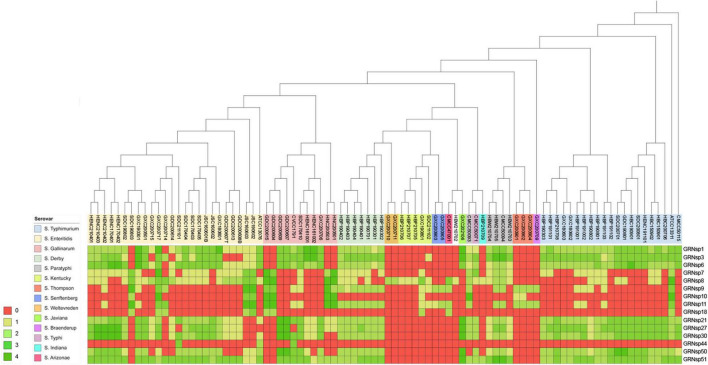
Lytic range of isolated phages. *Salmonella* strain susceptibility to phage infection. 0, no lytic plaque; +4, complete clear plaque.

In this study, a total of 15 *Salmonella* phages were isolated from 80 sewage effluent, sediment, and chicken manure samples. The host range of these phages was determined using 88 *S. enterica* strains collected from livestock and poultry farms in different geographical regions in China between 2015 and 2021. The GRNsp1, GRNsp3, GRNsp6, GRNsp21, GRNsp27, GRNsp30, GRNsp50, and GRNsp51 bacteriophages exhibited a relatively broad host spectrum and were able to infect a large proportion of *S.* Enteritidis and *S.* Typhimurium serovars to a varying degree (Figure 1). In particular, GRNsp51 had the widest host range, with a lysis rate of 80%, whereas GRNsp7, GRNsp8, GRNsp9, GRNsp10, and GRNsp11 had narrow host ranges but were able to increase the host spectrum of GRNsp51. Based on their host ranges and lytic capacities, we selected GRNsp6, GRNssp8, and GRNsp51 for further characterization.

### General Characterization of the Selected Phages

**FIGURE 2:**
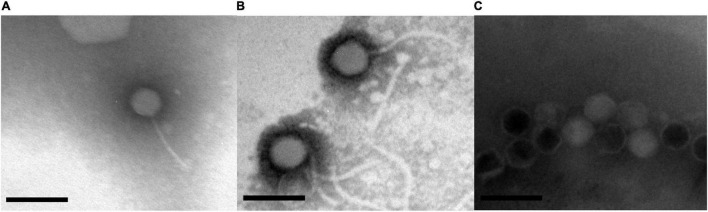
Morphology of three *Salmonella* phages. **(A)** GRNsp6, **(B)** GRNsp8, and **(C)** GRNsp51. Scale bar = 100 nm.

Morphological analysis using Transmission Electron Microscopy (TEM) revealed that GRNsp6, GRNsp8, and GRNsp51 all had tails and belonged to the order *Caudovirales*. GRNsp6 displayed some typical morphological features of *Siphoviridae* phages, with an icosahedral head (47 ± 1 nm diameter) and a non-contractile flexible tail (95 ± 3 nm; Figure 2A). Meanwhile, GRNsp8 was initially assigned to the *Siphoviridae* family due to the presence of a long, flexible, non-contractile tail (190 ± 9 nm) and a polyhedral head (61 ± 3 nm diameter; Figure 2B). GRNsp51 had a typical isometric head (52 ± 1 nm diameter) and an extremely short tail, indicating that this phage is a member of the *Podoviridae* family (Figure 2C). These morphological differences indicated these three phages are not identical and may have distinct host recognition mechanisms.

### Growth Characteristics and Thermal and pH Stability of Phages

**FIGURE 3:**
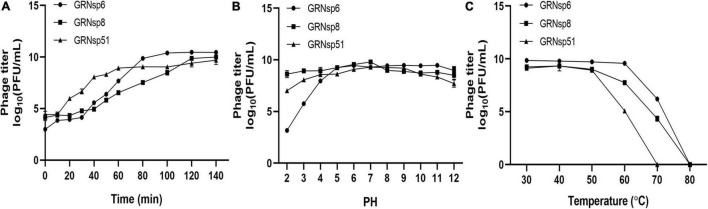
Phage growth curves and stability. **(A)** One-step growth curves of GRNsp6, GRNsp8, and GRNsp51 in *S.* Enteritidis ATCC13076 and ATCC13311 and *S.* Typhimurium CMCC50115, respectively, at an MOI of 0.01. Stability of GRNsp6, GRNsp8, and GRNsp51 with pH **(B)** and temperature **(C)**. Data represent the mean ± SD of three independent experiments was shown.

To determine the infection potential of GRNsp6, GRNsp8, and GRNsp51, we measured the one-step growth curves of each phage in TSB at a MOI of 0.01 (Figure 3A). GRNsp6 had a latency period of 20 min and an exponential growth period from 20 to 80 min, with an average burst size of 112 PFU/cell, whereas GRNsp8 had a burst size of 79 PFU/cell with latency period of 40 min. Unlike the other two phages, GRNsp51 had a short latency period of 10 min in *S*. Typhimurium with a burst size of 31 PFU/cell.

Next, we assessed the stability of the three phages to various pH conditions and elevated temperatures. GRNsp6, GRNsp8, and GRNsp51 maintained high titers (>7 log10 PFU/mL) at pH 4–12 over at least 12 h; however, the titer of GRNsp6 decreased significantly at pH 2–3 to 3.2 and 5.8 log10 PFU/mL, respectively (*p* < 0.05; Figure 3B). Notably, GRNsp8 was highly tolerant to a wide pH range (2–12), with high titers after 12 h at pH 2 and 12 (8.7 and 8.6 log10 PFU/mL, respectively). All three phages had > 50% viability from 30 to 50°C but were undetectable after exposure to 80°C for 60 min (Figure 3C). In particular, GRNsp51 showed relatively poor thermal tolerance and was completely inactive after incubation at 70°C for 60 min. The short latency period and excellent pH and thermal stability suggested these phages are good candidates for biocontrol.

### Phage Genome Analysis

**FIGURE 4:**
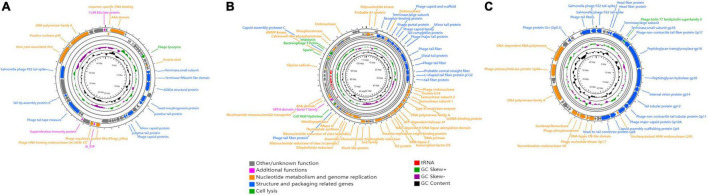
Schematic genome maps of **(A)** GRNsp6, **(B)** GRNsp8, and **(C)** GRNsp51. Circles are from inside to outside: G + C% content, GC skew plot, tRNA (dispensable), ORFs transcribed clockwise or counterclockwise denoted by specific colors according to their functional categories.

For phage therapy, it is essential to use virulent phages that lack genetic elements that might pose a threat to human health. To comprehensively determine the genetic characteristics of GRNsp6 (GenBank accession no. ON526838), GRNsp8 (GenBank accession no. ON526840), and GRNsp51 (GenBank accession no. ON526839), WGS was performed and their general features and genome were annotated (Table 3 and Supplementary Tables 1–3). GRNsp6, GRNsp8, and GRNsp51 have double-stranded DNA genomes composed of 43740, 111357, and 43461 bp, respectively, with GC contents of 49.5, 39.9, and 47.7%. The linear genome diagrams of the three phages are presented in Figure 4.

**TABLE 3:** Genomic properties of the three phages identified in this study and two other closely related phages (with the highest *E* value).

Categories	Name	Source	Genome size (bp)	Total ORFs	Identify	Query cover	GenBank acc. no.	References
Siphoviridae; Guernseyvirinae; Jerseyvirus	GRNsp6	Chicken farm, Jiangsu, China	43740	61				This study
	Salmonella phage vB_SenS-EnJE6	Sewage, Jilin, China	43129	67	93%	95%	MN336265.1	Direct submission
	Salmonella phage vB_SpuS_Sp4	Qingdao, China	43614	67	98%	98%	MH358359.1	Unpublished
Demerecviridae; Markadamsvirinae; Epseptimavirus	GRNsp8	Chicken farm, Wuhan, China	111357	191				This study
	Salmonella phage vB_SenS_SB6	Sewage, Ste Anne De Bellevu, QC, Canada	112311	165	93%	99%	MK809530.1	Unpublished
	Salmonella phage bombadil	Wastewater, Bethesda, MD, United States	109539	162	91%	99%	NC_048866.1	Direct submission
Autographiviridae; Molineuxvirinae; Zindervirus	GRNsp51	Chicken farm, Wuhan	43461	44				This study
	Salmonella phage UAB_Phi78	Barcelona, Espanya	43984	61	98%	97%	NC_020414.2	Bardina et al., 2012; Spricigo et al., 2013
	Salmonella phage BP12B	Sewage, Montreal, Canada	43602	51	94%	97%	KM366097.1	Direct submission

The GRNsp6 genome (Figure 4A) contained a total of 61 putative ORFs, of which 20 had annotated functions and the remaining 41 had other/unknown functions. The ORFs with annotated functions were categorized into three different functional modules related to structure and packaging (11 ORFs), cell lysis (1 ORF), and nucleotide metabolism and genome replication (8 ORFs). WGS analysis indicated that the nucleotide sequence similarity between the GRNsp6 genome and previously identified *Salmonella* phage vB_SenS-EnJE6 (GenBank: MN336265.1) of the *Jerseyvirus* genus within the *Siphoviridae* family was as high as 93%, with 95% coverage. Although the vB_SenS-EnJE6 genome has been submitted, the characteristics and clinical applications of this phage remain unclear.

As shown in Figure 4B, the GRNsp8 genome (≈111 Kbp) was approximately 2.5 times larger than that of GRNsp6 and its GC content (≈40%) was similar to that of representative phages from the *Epseptimavirus* genus (Cong et al., 2021). The GRNsp8 genome contained a total of 191 putative ORFs, among which 65 ORFs had annotated functions, as follows: 22 ORFs encoding structural proteins, 39 ORFs encoding proteins involved in nucleotide metabolism, DNA replication/repair/transcription, and 4 ORFs encoding cell lysis proteins. The remaining 100 ORFs had other/unknown functions. A total of 26 tRNA genes, including two pseudo-tRNA genes (tRNA*^Ala^* and tRNA*^Gly^*), were characterized and provided at least 19 codons (Supplementary Table 2). The presence of these tRNA genes would compensate for differences in codon and/or amino acid usage between the virus and host, reduce the dependence of the phage on the host, and improve fitness (Morgado and Vicente, 2019). In addition, tRNA^Met^ plays a pivotal role in phage translation initiation. BLASTN analysis classified GRNsp8 as a member of the *Epseptimavirus* genus within the family *Demerecviridae*. In addition, the GRNsp8 genome shared 99% nucleotide sequence similarity with the *Epseptimavirus Salmonella* phage vB_SenS_SB6 (GenBank: MK809530.1) with 93% coverage, whereas other phage genomes shared less than 93% coverage with GRNsp8.

The GRNsp51 genome contained no tRNAs and had 44 predicted ORFs (Figure 4C), among which 28 had annotated functions and 16 had other/unknown functions. BLASTN analysis revealed that the genomic sequence of GRNsp51 highly resembled the *Zindervirus*-like *Salmonella* phage UAB_Phi78 (98% coverage, 97% identity, GenBank: NC_020414.2), a phage component of a cocktail used to control *S.* Typhimurium (Bardina et al., 2012; Spricigo et al., 2013), as well as the directly submitted *Salmonella* phage BP12B (GenBank: KM366097.1) of the *Zindervirus* genus, with 97% nucleotide sequence similarity and 94% coverage.

No toxins, lysogeny-related genes (integrases, repressors, transposases, or excisionases), virulence factors, or AMR genes were detected in the GRNsp6, GRNsp8, or GRNsp51 genomes, indicating that they are virulent phages with the potential for biological safety applications. Whole genomic analysis revealed that the three phages belong to different genera (*Jerseyvirus*, *Epseptimavirus*, and *Zindervirus*, respectively) and display no nucleotide homology between their genomes.

### Phylogenetic Analysis

The terminase large subunit is a key component of DNA packaging machinery that is generally well conserved among tailed phages (Gambino et al., 2020). In addition, major capsid protein sequences have little or no evidence of horizontal swapping, meaning that they are more amenable to phylogenetic analysis (Sørensen et al., 2020). Therefore, we constructed phylogenies based on the terminase large subunit and major capsid proteins to evaluate the similarities in DNA packaging mechanisms and evolutionary homologies between the phages examined in this study and other similar phages.

**FIGURE 5:**
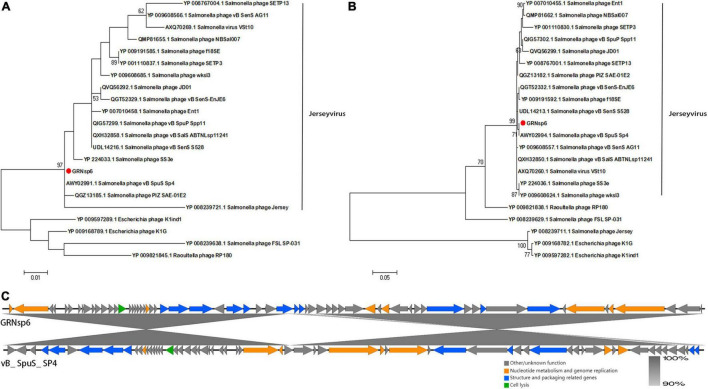
Genomic features of GRNsp6. Phylogenetic position of GRNsp6 within the *Guernseyvirinae* subfamily based on the amino acid sequences of the terminase large subunit **(A)** and major capsid protein **(B)**. Evolutionary analyses were conducted in Mega 7.0.14 using ClustalW alignment and the maximum likelihood method with 1000 bootstrap replicates. Bootstrap test percentages are displayed next to the branches. The phages investigated are marked with red circles. **(C)** Linear whole genome comparison of GRNsp6 and *Salmonella* phage vB_ SpuS_ SP4 using Easyfig v2.2.5. Genes with different functions are denoted by specific colors. Regions of nucleotide homology are shaded with gray lines and are > 90% similar.

Phylogenetic analysis based on major capsid proteins and terminase large subunits revealed that GRNsp6 shared the highest similarity with *Salmonella*_ phage_ vB_ SpuS_ SP4 (Figures 5A,B; Refseq Accession: AWY02991.1, AWY02994.1). Genome comparison between GRNsp6 and vB_ SpuS_ SP4 (GenBank: MH358359.1) indicated that the main difference between the genomes of these phages was in major capsid protein-related genes (Figure 5C), whose mutations can cause abnormalities in phage assembly. Consistent with the BLASTN search results, phylogenetic analysis revealed that GRNsp6 can be classified as a member of *Jerseyvirus* genus in the *Siphoviridae* family.

**FIGURE 6:**
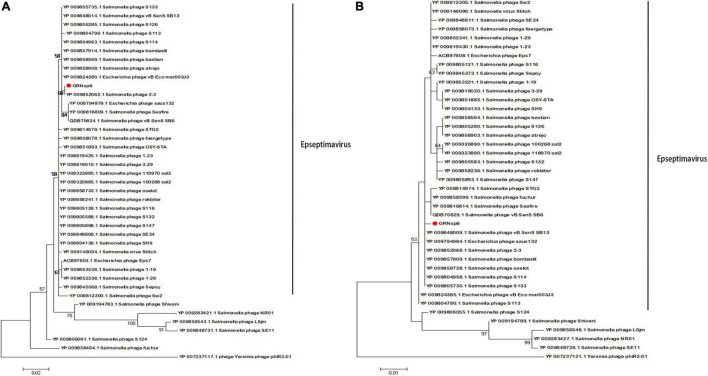
Phylogenetic analysis of GRNsp8. Phylogenetic tree based on the sequences of major capsid proteins **(A)** and terminase large subunits **(B)** from 41 phages.

The sequence of the gene encoding the major capsid protein of GRNsp8 formed a subclade with *Salmonella* phage 2-3 (Figure 6A; Refseq Accession: YP_009852068.1), a ‘T5-like phage’ morpho-type of the *Epseptimavirus* genus within *Demerecviridae* family. The sequence of the gene encoding the terminase large subunit of GRNsp8 was located in a single clade and was highly homologous with that of *Salmonella*_phage_vB_SenS_SB13 (Refseq Accession: YP_009848009.1), *Escherichia*_phage_saus132 (Refseq Accession: YP_009794984.1), and *Salmonella* phage 2-3 (Figure 6B).

**FIGURE 7:**
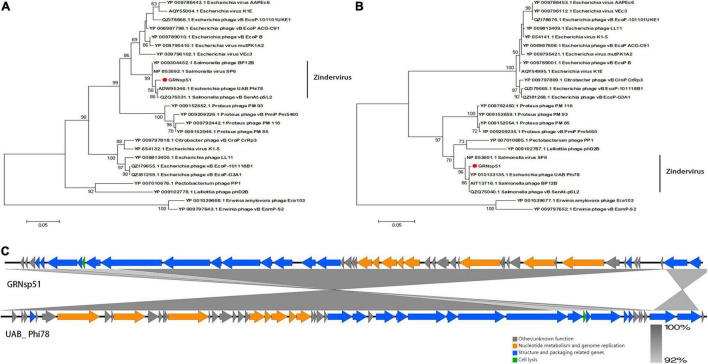
Genomic features of GRNsp51. Phylogenetic analysis between GRNsp51 and 25 known phages within the *Molineuxvirinae* subfamily according to their major capsid protein **(A)** and terminase large subunit **(B)** sequences. **(C)** Linear whole genome comparison of GRNsp51 and Salmonella phage UAB_Phi78 using Easyfig v2.2.5. Genes with different functions are denoted by specific colors. Regions of nucleotide homology are shaded with gray lines and are > 92% similar.

Phylogenetic analysis based on the major capsid and terminase large subunit (Figures 7A,B) revealed that GRNsp51 had a close relationship with T7-like bacteriophage UAB_ Phi78 (Refseq Accession: ADW95246.1, YP_010133135.1), vB_ Senat-psl2 (Refseq Accession: QZQ75031.1, QZQ75040.1), BP12B (Refseq Accession: YP_009304452.1, AIT13716.1), and SP6 (Refseq Accession: NP_853592.1, NP_853601.1). Notably, GRNsp51 was located in a single clade that was distinct from all other phages of the *Zindervirus* genus available in the International Committee on Taxonomy of Viruses (ICTV^5^) (Lefkowitz et al., 2018) and NCBI. Thus, GRNsp51 could be regarded as a new species of *Zindervirus*. Genome comparison between GRNsp51 and homologous phage UAB_ Phi78 revealed that the major differences between the two genomes were the regions of head fiber, tail fiber proteins for host recognition, as well as the terminase large subunit for DNA packaging machinery, implying that these phages may have different host ranges or different packaging mechanisms (Figure 7C). The phylogenetic diagram indicated that GRNsp6 and GRNsp8 were closely associated with *Jerseyvirus* genus and *Epseptimavirus* genus, respectively, and that GRNsp 51 is the new member of the *Zindervirus* genus.

### Phage Titers in Chicken Serum and Intestinal Tracts Over Time

**FIGURE 8:**
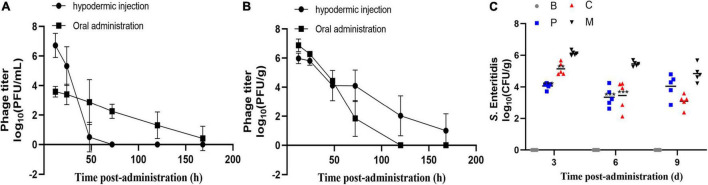
Residence time and efficacy of the phage cocktail in chickens. Abundance of single dose phage cocktail (10^10^ PFU/mL) in the serum **(A)** and cecum **(B)** of chickens at different times. Data represent the mean ± SD (*n* = 4). **(C)** Effect of phage therapy on the viability of *S.* Enteritidis ATCC13311 in the cecum on days 4, 7, and 10 (log10 CFU/g). Group C, phages were administered concurrently with bacterial infection; Group P, phages were administered one day before infection; Group M, bacterial infection only; Group B, PBS only control group. *p < 0.05, **p < 0.01, ***p < 0.001.

Previous studies have reported that the administration of phages at titers of around 10^9^ PFU/mL can yield notable therapeutic efficiency (Gigante and Atterbury, 2019; Bao et al., 2020). Therefore, we measured the abundance of 0.1 mL phage cocktail (10^10^ PFU/mL) in the intestine and serum of chickens over time following a single oral treatment or injection. As shown in Figure 8A, oral administration resulted in extraordinarily low serum phage titers (≤ 10^4^ PFU/mL at 12 h); however, the maintenance time was relatively long, with phages still detectable in serum after 168 h. In the cecum (Figure 8B), phage titers remained high (10^7^ PFU/g) 12 h after administration but decreased rapidly to ≤10^3^ PFU/g at 72 h. After injection (Figures 8A,B), the phages rapidly reached titers of 10^7^ PFU/mL and 10^6^ PFU/g in the blood and cecum after 12 h, respectively. Phage titers then decreased with time following administration and decreased faster in the blood than in the cecum. After 120 h, no phages were detected in the serum but phages were still detected in the cecum until the end of the experiment (data not shown). These results suggested that either oral or injected phages were able to enter the cecum and blood. In order to avoid severe stress response with intraperitoneal injection, we employed oral administration.

### Biocontrol Efficacy of the Bacteriophage Cocktail Against Multidrug-Resistant Salmonella Enteritidis in Chickens

Next, we investigated the ability of the phage cocktail to reduce the *Salmonella* population in chickens (Figure 8C). As expected, no *Salmonella* or corresponding phages were recorded in the control group. Compared to group M, the *Salmonella* concentration in group P was reduced by 2.1 log10 (*p* = 0.0003) and 1.8 log10 (*p* = 0.0005) in the cecum on days 4 and 7 post-infection, respectively, and by 1.6 log10 (*p* > 0.05) on day 10 (Figure 8). A similar change in *Salmonella* concentration was observed in group C, with a 0.9 log10 (*p* = 0.0011), 1.6 log10 (*p* = 0.0005), and 1.9 log10 (*p* > 0.05) reduction on days 4, 7, and 10 post-infection, respectively. Moreover, no significant difference was observed between groups C and P (*p* > 0.05). The phage concentration in the cecum ranged from 10^6^ to 10^7^ PFU/g throughout the experiments. The phage cocktail in this study showed promising effects in reducing the *S.* Enteritidis counts under laboratory conditions.

### Intestinal Inflammatory Factor Expression

**FIGURE 9:**
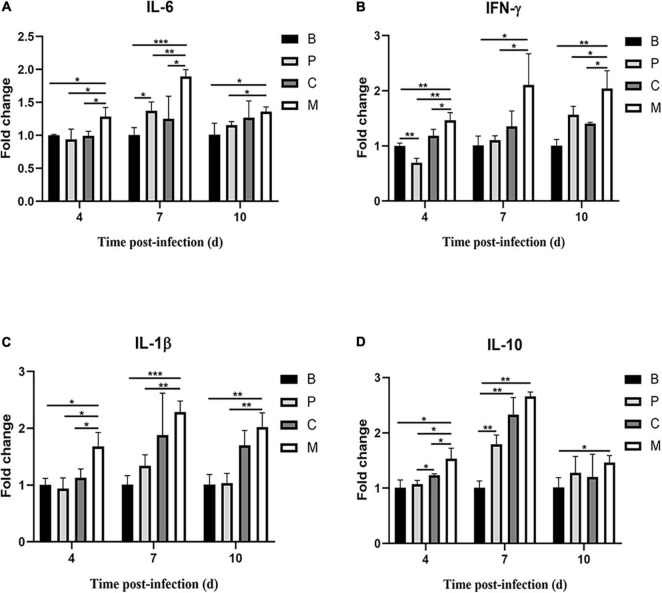
Relative cytokine expression in the small intestine of chickens over time. **(A)** Interleukin-6 (IL-6), **(B)** interferon-γ (IFN-γ), **(C)** interleukin-1β (IL-1β), **(D)** interleukin-10 (IL-10). Data represent the mean ± SD (*n* = 5). *p < 0.05, **p < 0.01, ***p < 0.001.

Finally, we measured the expression of genes encoding inflammatory factors in intestines of chickens treated with the phage cocktail. Compared to the control group, chickens in the M group had increased IL-6, IFN-γ, IL-1β, and IL-10 mRNA expression on days 4, 7, and 10 post-infection (*p* < 0.05, Figure 9). IL-6, IL-1β, and IL-10 mRNA expression did not differ significantly (*p* > 0.05) in chickens treated with the bacteriophage cocktail the day before infection with *S.* Enteritidis (Group P) on days 4 and 10 compared to the control group; however, IL-6 and IL-10 mRNA expression were increased (*p* < 0.05) and IL-1β expression was slightly increased (*p* = 0.08) on day 7. Similar trends in cytokine mRNA expression were observed in chickens treated with the phage cocktail alongside infection (Group C) and in those given the cocktail the day before infection. Dietary supplementation with phages significantly reduced IL-6, IFN- γ, IL-1 β, and IL-10 mRNA expression on day 3 post-infection (*p* < 0.05) compared to group M. In this study, we found that *Salmonella* infection caused increased expression of pro-inflammatory factors IL-6, IFN- γ, and IL-1 β in chickens, whereas either pre – or simultaneous supplementation of phage reversed this phenomenon.

## Discussion

Human and animal infections caused by *Salmonella* have become a critical issue worldwide due to the emergence of AMR. Bacteriophages are considered the most promising alternative to antibiotics for pathogen control and several commercial phage products targeting *Salmonella* spp. have been applied in the poultry industry, including Salmofresh ™ (Intralytix. Inc., Baltimore, MD, United States) and Salmonelex ™ (Micreos Food Safety BV, Wageningen, Netherlands). However, these strategies are limited by the fact that one phage can only infect a few target *Salmonella* spp. A critical feature of phage therapy is therefore considered to be a wide host range, which can be achieved by using a cocktail composed of multiple phages. In this study, we isolated a total of 15 phages with high lytic capacity against MDR *Salmonella* and further characterized the biological, genomic, and evolutionary properties of three broad-spectrum virulent phages (GRNsp6, GRNsp8, GRNsp51) against *S*. Enteritidis and *S.* Typhimurium, which are the most frequently isolated serovars from foodborne salmonellosis outbreaks, and other *Salmonella* serovars such as *S.* Pullorum, which often occurs in poultry farms in China (European Food Safety Authority [EFSA], 2010; Barrow and Freitas Neto, 2011; European Food Safety Authority and European Centre for Disease Prevention and Control, 2021).

We assayed the ability of a cocktail consisting of the GRNsp6, GRNsp8, and GRNsp51 phages to reduce the concentration of *Salmonella* in the intestinal tract of chickens. Eighty *Salmonella* strains were isolated from clinical poultry and swine samples, as well as environmental sewage, and included a variety of serovars associated with *Salmonella* infections in humans, such as Enteritidis, Typhimurium, 1,4,[5],12:i:-, Derby, Weltevreden, Senftenberg, Thompson, Kentucky, Indiana, Arizona, Javiana, and Braenderup. As shown in Supplementary Table 4, these *Salmonella* isolates were highly resistant to SF (100%), S (95%), AMP (68.8%), NA (67.5%), CIP (66.3), K (61.3%), CN (53.8%), and TE (52.5%). In addition, each isolate was resistant to at least one class of antimicrobial (Table 1). The three isolated phages displayed distinct host spectrums against these *Salmonella* species, with particularly strong lytic activity against *S.* Enteritidis and *S.* Typhimurium and significant growth inhibitory effects against other serovars such as *S.* Gallinarum, *S.* Javiana, *S.* Derby, *S.* Typhi, and *S.* Paratyphi. Among the 15 newly isolated phages, GRNsp51 displayed the widest host range and lysed 80% of *Salmonella*. Strains representing the *S.* Senftenberg, *S.* Thompson, *S.* Braenderup serovars were not lysed by GRNsp6, GRNsp8, and GRNsp51 but were lysed by other isolated phages, probably due to a lack of appropriate receptors or other bacteria-related resistance mechanisms. One-step growth analysis further indicated that GRNsp6, GRNsp8, and GRNsp51 possessed relatively short latency periods and large burst sizes, indicating that they are capable of rapid infection and proliferation. In addition, all three phages exhibited relatively high thermostability and survivability over a wide pH range, which are essential properties for biocontrol phages as they enable them to survive in harsh environments such as the gastrointestinal tract. The observed variation in host range and morphology between the three phages suggests that they may recognize distinct host receptors during infection (Shin et al., 2012). Consistently, previous studies have indicated that using a combination of phages recognizing distinct host receptors may delay the emergence of phage-resistant mutants (Bardina et al., 2012; Chan et al., 2013). Although this was not assessed in our study, we found that the combined use of these three phages increased the host spectrum of each individual phage to enable the efficient infection of multiple *S. enterica* serovars and strains.

Whole-genome analysis confirmed that all three phages lacked genes that may be involved in lysogenization, virulence, and AMR, suggesting that these three phages are virulent and safe. Thus, GRNsp6, GRNsp8, and GRNsp51 could be appropriate candidates for phage therapy and feed additive purposes. The genome of GRNsp8 contained 26 tRNA genes. tRNA genes are not rare in *Salmonella* phages (Morgado and Vicente, 2019; Al-Shayeb et al., 2020; Duc et al., 2020), and the total number of tRNA genes has been reported to positively correlate with genome length (Morgado and Vicente, 2019; Al-Shayeb et al., 2020). Although the GRNsp8 genome contains many tRNAs, it only around 111 Kbp. Previous reports have indicated that tRNA genes are considered clustered if their tRNA gene density is ≥ 2 tRNA/KB (Bermudez-Santana et al., 2010), which facilitates genome compaction. Therefore, tRNA clustering was present in the GRNsp8 genome. Notably, tRNAs can participate in lateral gene transfer and can enhance protein synthesis and infectivity in virulent bacteriophages to increase their virulence. Cluster analysis based on the major capsid proteins and the terminase large subunit indicated a similar grouping pattern to the whole genomes, reinforcing their utility as marker genes for genetic relevance. Phylogenetic analysis revealed GRNsp6 is a member of the *Jerseyvirus* genus in the *Siphoviridae* family, while GRNsp8 is a member of the *Epseptimavirus* genus in the *Demerecviridae* family and GRNsp51 is a new member of the *Zindvirus* genus within the *Podoviridae* family.

Chicken models provide extremely valuable information regarding the efficacy of phage therapy and drive important advances in phage research. In this study, we investigated the abundance and maintenance time of phages in chicken blood and intestines following intraperitoneal injection or oral administration with a high-titer single dose of the phage cocktail. Notably, oral administration yielded a phage concentration of approximately 10^7^ PFU/g in the cecum and phages were also detected in the blood (≤10^4^ PFU/mL). Consistently, many studies have confirmed that phages administered orally can translocate into the whole body through the intestinal wall (Górski et al., 2006). When the phage cocktail was administered to chickens either before or simultaneously with *S.* Enteritidis, the concentration of *Salmonella* was significantly decreased; however, resurgence was observed after 10 days. At the end of the experiment, the inability of the phage cocktail to completely eliminate the *Salmonella* concentration in the gut was likely due to the emergence of phage-resistant mutant strains. The loss of bacterial receptors and inherent restriction modification systems or CRISPR-Cas can affect phage infectivity (Maffei and Shaidullina, 2021). However, it has been suggested that the emergence of resistant strains could be slowed by the use of phage cocktails that recognize independent receptors (Hyman and Abedon, 2010; Chan et al., 2013). In this study, we used high concentrations of phages recognizing different receptors to minimize this effect. Although the phages were not able to completely clear the *Salmonella* infection, the cocktail was able to maintain the infection at a low level, as confirmed by the levels of inflammatory factors detected. In the future, we will explore the mechanism of phage-host interactions to provide guidance for the scientific application of phages.

In summary, we isolated and characterized three novel *Salmonella* phages exhibiting high lytic activity against MDR *Salmonella* strains from multiple serovars, as well as excellent biological and safety characteristics. Furthermore, we found that a cocktail prepared using these three phages was able to successfully control *Salmonella* infection *in vivo*.

## Data Availability Statement

The datasets analyzed in this study are available NCBI (BioProject ID: PRJNA844535).

## Ethics Statement

The animal study was reviewed and approved by the Research Ethics Committee of Huazhong Agricultural University.

## Author Contributions

XW and YL contributed to the concept and method of the manuscript. YL obtained the manuscript data and drafted the manuscript. PL, HZ, XY, and XJ analyzed or interpreted the manuscript data. XJ, DS, and XW reviewed the manuscript. All authors read and approved the published version of the manuscript.

## Conflict of Interest

The authors declare that the research was conducted in the absence of any commercial or financial relationships that could be construed as a potential conflict of interest.

## Publisher’s Note

All claims expressed in this article are solely those of the authors and do not necessarily represent those of their affiliated organizations, or those of the publisher, the editors and the reviewers. Any product that may be evaluated in this article, or claim that may be made by its manufacturer, is not guaranteed or endorsed by the publisher.

## Footnotes

1
https://sistr-app.herokuapp.com/

2
http://vogdb.org

3
https://www.ncbi.nlm.nih.gov/

4
https://phaster.ca/

5
http://ictv.global

## Abbreviations

GD: Guangdong
GX: Guangxi
HEN: Henan
SD: Shandong
JS: Jiangsu
HB: Hubei
HN: Hunan.
